# Implementation of The First Breastfeeding in Warsaw's Maternity Hospitals

**DOI:** 10.34763/devperiodmed.20192304.209215

**Published:** 2021-01-29

**Authors:** Monika Salamończyk, Anna Łozińska-Czerniak, Ewa Dmoch-Gajzlerska, Magdalena Bednarczyk

**Affiliations:** 1 Department of Gynecology and Obstetrics Didactics, Faculty of Health Sciences, Medical University of Warsaw, Warsaw Poland; 2 Neonatology and Neonatal Intensive Care Clinic, Institute of Mother and Child, Warsaw, Poland

**Keywords:** breastfeeding, skin-to-skin contact, newborn, karmienie piersią, kontakt, skóra do skóry", noworodek

## Abstract

**Introduction:**

Birth is associated with the loss of the comfortable intrauterine environment and the beginning of life in the external environment. The newborn undertakes basie life functions in an environment of stimuli to which his nervous system is particularly sensitive. Some fundamental methods that facilitate adaptation to extrauterine life are skin-to-skin contact and the first breastfeeding.

**Aim:**

The aim of the study was to assess the provision of the first breastfeeding after natural delivery in Warsaw's medical facilities.

**Materiał and method:**

The method of direct observation was used in the study. The research material was collected using the authored observation sheet designed for the purposes of the study. The tests were carried out in 11 Warsaw obstetric facilities with varying degrees of referentiality, in which the management agreed to conduct the study. It was ensured that the data collected would be presented anonymously. The research began in January 2016 and was completed in December 2017.

**Results:**

During the direct contact of the mother with the child, in 97.37% of the cases the newborn was attached to the mother’s breast. In 25.01°% of the cases, the duration of the first breastfeeding was over 30 minutes, and in the same number offeedings it ranged from 21-30 minutes. The shortest duration of feeding from 1-5 minutes was observed in 5.58% of the cases.

**Conclusions:**

In most cases, the first breastfeeding took place within 2 hours of birth. The initiation of breastfeeding after natural delivery took place during the mother’s first contact with the child. In more than half of the cases, the first feeding lasted as long as the specialists recommend - over 20 minutes.

## Streszczenie

**Wstęp:**

Narodziny związane są z utratą komfortowego środowiska wewnątrzmacicznego i rozpoczęciem życia w środowisku zewnętrznym. Noworodek podejmuje samodzielnie podstawowe funkcje życiowe w środowisku bodźców na które szczególnie wrażliwy jest jego układ nerwowy. Metodami ułatwiającymi adaptację do życia pozamacicznego jest kontakt„skóra do skóry” oraz pierwsze karmienie piersią.

**Cel:**

Celem pracy była ocena zapewnienia pierwszego karmienia piersią po porodach drogami natury w warszawskich placówkach medycznych.

**Materiał i metoda:**

W badaniu wykorzystano metodę obserwacji bezpośredniej. Materiał badawczy gromadzony był za pomocą autorskiego arkusza obserwacji skonstruowanego na potrzeby badania. Badania przeprowadzono w 11 warszawskich placówkach położniczych o różnym stopniu referencyjności, w których dyrekcja wyraziła zgodę na przeprowadzenie badania. Zapewniono, iż pozyskane dane zostaną przedstawione anonimowo. Badania rozpoczęto w styczniu 2016 roku, a zakończono w grudniu 2017 roku.

**Wyniki:**

W czasie trwania bezpośredniego kontaktu matki z dzieckiem w 97,37% przypadków przystawiono noworodka do piersi matki. W 25,01% przypadków czas trwania pierwszego karmienia piersią wynosił powyżej 30 minut, tyle samo było karmień wynoszących od 21-30 minut. Najkrócej trwające karmienia od 1-5 minut zaobserwowano w 5,58% przypadków.

**Wnioski:**

W większości przypadków pierwsze karmienie piersią przebiegało w czasie do 2 godzin od urodzenia. Inicjacja karmienia piersią po porodach drogami natury odbywała się w czasie realizacji pierwszego kontaktu matki z dzieckiem. W ponad połowie przypadków pierwsze karmienie trwało tyle ile zalecają specjaliści – ponad 20 minut.

## Introduction

The first hours after birth are considered to be the most important stages in human development. They are defined as a decisive time in life. The newborns nervous system is particularly sensitive to negative stimuli. The recommended method of facilitating adaptation to extrauterine life is skin-to-skin contact and the first breastfeeding [1].

In the 1980s, the World Health Organization (WHO) issued recommendations highlighting the need for skin-to-skin contact. The “Child Friendly Hospital” program was created, whose overall goal is to promote activities supporting and promoting breastfeeding in healthcare facilities in the form of “10 steps to successful breastfeeding.” In Poland, the recommendations in the Regulation of the Minister of Health on the organizational standard of perinatal care are currently in force, regulating postpartum care procedures including 8 out of the 10 steps. They include mother-child contact, which consists of placing a naked child on the mothers chest/stomach before cutting the umbilical cord and the first breastfeeding of the newborn baby after birth. The mother’s contact with the child may be interrupted only for medical reasons in the event of a threat to the life or health of the mother and/or child [2, 3, 4].

Attaching the baby to the breast has an impact on the course of lactation and its duration. Early breast sucking begins the production of food and promotes lactogenesis by secretion of prolactin (which is released from the anterior pituitary as a result of the stimulus of sucking) and oxytocin (from the posterior pituitary). Many studies have shown a relationship between skin-to-skin contact with the mother together with early initiation of the first feeding and exclusive breastfeeding at discharge from the hospital and up to three or even six months after birth [5, 6, 7, 8]. The test results also apply to cesarean sections. In the group where the first breastfeeding began early, newborns were exclusively fed with mother’s milk up to the age of three months [9].

The first feeding during contact with the mother is of particular importance at many levels, both for mother and baby. The advantages resulting from early breastfeeding are:

– beneficial effect on neurobehavioral processes - newborns cry less, calm down, show more of flexion movements compared to extension movements [10],
– creating conditions for proper thermoregulation and eliminating thermal stress after birth - the mother and child’s heat synchronize with each other (when the child’s temperature decreases, the mother’s temperature increases) [11],
– starting parent-child interaction in the area of mental relations, minimizing the stress associated with the act of giving birth by being comforted in the mother’s arms, building bonds with the child through eye contact, kissing, stroking [11],
– higher blood glucose levels in newborns in the first hours of life fed during “skin-to-skin” contact [12],
– undergoing the first feeding allows babies to continue the functional and morphological maturation of the gastrointestinal tract and other organs and provides anti-infective protection by ingredients found in the swallowed colostrum [13],
– in the case of children born by caesarean section who are in good general condition, their hospitalizations in the intensive care unit are less frequently observed [14],
– improvement of the mother’s self-esteem in terms of independent childcare, a sense of self-confidence and satisfaction with childbirth [6],
– reduction of anxiety in the mother related to the well-being of the newborn in case of cesarean section [15],
– a soothing and calming effect on the mother, reducing her tension, lowering blood pressure and a positive effect on her bond with the child - the action of endogenous oxytocin [16],
– faster and more effective contraction of the uterus muscle (a beneficial phenomenon that reduces the amount of blood lost after delivery by the mother) - this effect is associated with the endogenous release of oxytocin during breast sucking [17].

A significant benefit resulting from early breastfeeding is the colonization of the digestive tract of the newborn by the mother’s commensal microbiota. An important role is played by colostrum - the first food. Compared to mature foods, it contains less fat and lactose, more vitamin A and protein, which prevents severe neonatal jaundice by speeding up meconium excretion. It also contains bacteria of the genera Lactobacillus and Bifidobacterium and olisaccharides that affect their development. Colostrum contains high levels of antiinflammatory factors and cytokines and antibodies (Class A secretory immunoglobulin) [18, 19].

## Aim

The aim of the study was to assess the provision of the first breastfeeding after natural delivery in Warsaw’s medical facilities in the light of the current Perinatal Care Organization Standard and WHO guidelines regarding the mother’s first contact with the child.

## Material and method

The method of direct observation was used in the study. The research material was collected using the authors’ observation sheet designed for the purposes of the study.

The sheet contained:

– general data: date of observation, place of observation, number of observations in a given hospital, degree of center reference,
– data on delivery: week of completion of pregnancy, type of delivery, the newborn’s condition after birth, Apgar score at the first and fifth minutes of life,
– data on the first feeding: time to start feeding, duration of feeding, person attaching the newborn to the breast.

Criteria for inclusion in the study:

– written consent of the hospital director to participate in the study,
– location of the hospital in Warsaw,
– location of the hospital in the annual statistical report for 2015,
– getting 8, 9 or 10 points according to the Apgar score in the first and fifth minute of life. Exclusion criteria for the test:
– lack of consent of the hospital management to participate in the study,
– location of the hospital outside Warsaw,
– getting a newborn with from 7 to 0 points on the Apgar score in the first minute of life,
– condition of the newborn baby preventing the mother from making the first contact with the child confirmed by a neonatologist.

The tests were carried out in 11 Warsaw obstetric facilities with varying degrees of referentiality, in which the management agreed to conduct the study. It was ensured that the data collected would be presented anonymously. The research began in January 2016 and was completed in December 2017.

The analysis included 304 observations. The number of observations in a given hospital was determined proportionally on the basis of the annual summary of the number of deliveries in Warsaw in 2015. The persons conducting the study were trained how to run the observations before the start of the study and were not employed in the facilities where the study was conducted. The observations were carried out from the moment the child was born until the end of the fourth delivery period. The observers recorded the results obtained on an observation sheet.

## Results

The studies were carried out in hospitals with varying degrees of reference. There were 6 hospitals with the 3rd reference level - 176 observations (57.89%), 4 hospitals had the 2nd reference level -117 observations (38.49%), 1 hospital had the 1st reference level, and 11 observations were made there (3.62%) (Table I).

Analysis was conducted of 304 observations of deliveries completed by natural labor. The shortest gestation was 35 weeks and the longest 41 weeks. The average time of completion of pregnancy in the study group was 39.11 ± 1.12 (Table II).

In the group of newborns that was observed, 97.70% were mature, healthy children, 2.30% were prematurely born in good general condition (Table III).

**Table I:** The number of deliveries observed broken down by the reference levels of the hospitals where they took place. Tabela I. Liczba porodów poddanych obserwacji w szpitalach z podziałem na stopnie referencyjności.

	N	%
**3rd reference level/III *stopień referencyjności***	176	57.89
**2nd reference level/ll *stopień referencyjności***	117	38.49
**1st reference level// *stopień referencyjności***	11	3.62

**Table II:** Week of gestation at delivery in the patients observed. Tabela II. Tydzień ukończenia ciąży u obserwowanych pacjentek.

	N	Mean *Średnia*	SD *Odchylenie standardowe*	Median *Mediana*	Q1	Q3	Minimum	Maximum
**Week at delivery** ***Tydzień ukończenia ciąży***	304	39.11	1.12	39.00	38.00	40.00	35.00	41.00

**Table III:** Neonatal maturation characteristics after birth. Tabela III. Charakterystyka dojrzałości noworodków po urodzeniu.

	N	%
**Mature, healthy baby** ***Noworodek dojrzały, zdrowy***	297	97.70
**A premature baby, healthy** ***Noworodek przedwcześnie urodzony, zdrowy***	7	2.30

**Table IV:** The number of points according to the Apgar scale obtained by a newborn baby in the first minute of life. Tabela IV. Liczba punktów wg skali Apgar uzyskana przez noworodka w 1. minucie życia.

	N	%
**10 points/10 *punktów***	263	86.52
**9 points/9 *punktów***	24	7.89
**8 points/8 *punktów***	17	5.59

**Table V:** The number of points according to the Apgar scale obtained by a newborn in the 5th minute of life. Tabela V. Liczba punktów wg skali Apgar uzyskana przez noworodka w 5. minucie życia.

	N	%
**10 points/*10 punktów***	302	99.34
**9 points/9 *punktów***	2	0.66
**8 points/8 *punktów***	0	0.00

**Table VI:** Newborn attachment to the breast within 2 hours of birth. Tabela VI. Przystawienie noworodka do piersi w ciągu 2 godzin od urodzenia.

	N	%
***Yes/Tak***	296	97.37
**No/*Nie***	8	2.63

**Table VII:** The person who attached the newborn to the breast. Tabela VII. Osoba, która przystawiła noworodka do piersi.

	N	%
**Midwife/Po/ożno**	135	44.40
**Student/*Studentka***	107	35.20
**The newborn attachedherself to the breast** ***Noworodek samodzielnie*się przystawił do piersi**	4	1.32
**Mother after instructionby midwife** ***Matka po instruktażuprzez położną***	18	5.92
**Mother without instruction** ***Matka bez instruktażu***	32	10.53
**Not applicable/*Nie dotyczy***	8	2.63

The analysis of data on the number of points on the Apgar score obtained in the first minute of a child’s life showed that 86.52% of the newborns scored 10 points, 7.89% were rated 9 points, and 5.59% scored 8 (Table IV).

In the 5th minute of life, 99.34% of newborns obtained 10 points on the Apgar score, 0.66% were rated 9 (Table V).

During the direct contact of the mother with the child in 97.37% of the cases the newborn was attached to the mother’s breast. In 2.63% of the cases, the newborn was not attached to the breast (Table VI).

In 44.40% of the cases the newborn was attached to the breast by a midwife, 35.20% a midwife student, and in 10.53% a mother without instruction. In 5.92% of cases, the attachment took place after the midwife’s instruction. Some cases of the newborns independent attachment to the breast were noticed. These accounted for 1.32% of the cases. The “Not applicable” group are cases in which the newborn was not attached to the breast (Table VII).

The duration of the first breastfeeding was analyzed. In 25.01% of the cases it lasted over 30 minutes, in the same number of feedings it ranged from 21-30 minutes. The shortest feeding time from 1-5 minutes was observed in 5.58% of the cases (Table VIII).

## Discussion

Breastfeeding (in exceptional cases a bottle with breast milk) is the proper way to feed newborns and young children. The superiority of breast milk over dairy formula based on cow’s milk was recognized by WHO, UNICEF *(United Nations International Childrens Emergency Fund)*, AAP *(American Academy of Pediatrics)*, ESPGHAN *(The European Society for Paediatric Gastroenterology Hepatology and Nutrition)* and EFSA *(European Food Safety Authority)*. As early as 1974, WHO announced the value of exclusive breastfeeding in the first months of life. Currently, many institutions and organizations on a global and local scale are undertaking activities to support this diet [18, 20, 21, 22]. In 1990, the initiative “Child Friendly Hospital” was created, whose main task is to disseminate practices that promote and support breastfeeding in healthcare facilities in the form of “10 steps to successful breastfeeding.” Their content (with minor modifications) was unchanged. In 2017, WHO published a new edition of the document ‘Protection, promotion and support of breastfeeding in obstetric and neonatal care facilities’, which contained the updated TO steps to successful breastfeeding’. The current document has been divided into two parts. The first contains management procedures for successful breastfeeding, the second gathers key clinical practices for breastfeeding. The fourth step provides tips to “enable mother and baby to have uninterrupted” skin-to-skin “contact as soon as possible after delivery and help her initiate breastfeeding as soon as possible, help a woman start breastfeeding within half an hour of having a baby” [20].

**Table VIII:** Duration of the first breastfeeding. Tabela VIII. Czas trwania pierwszego karmienia piersią.

	N	%
**1-5 minutes/1-5 *minut***	17	5.58
**6-10 minutes/6-10 *minut***	54	17.76
**11-20 minutes/21-20 *minut***	73	24.01
**21-30 minutes/21-30 *minut***	76	25.01
**Over 30 minutes/*Powyżej 30 minut***	76	25.01
**Not applicable/*Nie dotyczy***	8	2.63

Immediate skin-to-skin contact and early initiation of breastfeeding are two closely linked aspects that must occur together to achieve optimal benefits [2, 5, 9, 12, 23, 24, 25, 26]. Such contact stimulates the newborn’s search reflex, which helps him find the breast and then suck it, thus initiating food production and supporting lactogenesis [16]. The first feeding should last without interruption until natural completion. If the newborn actively sucked the breast immediately after birth, he or she may not show the desire to suck, nor indicate hunger for the next 12 hours [5, 27].

In our own research, it was noted that during the direct contact of the mother with the child, the newborn was attached to the mother’s breast in most cases (97.37%). In 25.01% of our cases, the first feeding lasted over 30 minutes and the same number of feedings took from 21 to 30 minutes. Only in 5.58% of the cases was it short - from 1 to 5 minutes. Such a well-initiated first feeding indicates that the majority of hospitals implement the fourth step of the “10 steps ...” although according to KUKP1awarding the title, KUKP confirms that a given health care facility carries out all the” 10 Steps ... ,„ is comparable with decorated facilities from around the world, and its course of conduct serves the best interests of the child and mother, enabling breastfeeding. At the same time, the „International Marketing Code for Feminine Milk Replacement Products” is fully respected in such an institution, giving mothers the chance to make a decision to feed their child based on modern medical knowledge, not on advertising. “[28] (the *Breastfeeding Dissemination Committee}* only four hospitals (out of the eleven in which the studies were conducted) were awarded the title of a “Child Friendly Hospital” (three hospitals received a re-evaluation). In Poland, only ninety hospitals have this title [28]. According to SOOO (the *Perinatal Care Organization Standard}*, the task of medical staff is to encourage the mother to recognize the baby’s readiness to suck and to offer her help if she needs it. You should also check the features of good attachment and the selection of the optimal position [4]. In the study, in 85.52% of the cases analyzed, the mother received help in attaching the newborn to the breast. In 10.53%, the mother initiated lactation on her own, without an instructor. Independent cases of newborn attachment to the breast were observed in 1.32% cases. Many studies have shown a relationship between skin-to-skin contact with the mother and feeding time up to three or even six months after birth [5, 8, 9, 29]. Data on the start of breastfeeding in different regions of the world vary and depart from WHO recommendations. Under half of all newborns in the world are breastfed during the first hour of life [30]. In a few European countries, i.e. Denmark and Norway, 90% start such feeding, in several countries (United Kingdom, Belgium) the relevant number ranges from 60-70% [22]. Many women stop breastfeeding early or think that they do not have enough food, therefore early, properly implemented breastfeeding during the first contact with the mother is of great importance for the success of breastfeeding.

## Conclusions

1. In most cases, the first breastfeeding took place within 2 hours of birth.
2. The initiation of breastfeeding after natural childbirth took place during the implementation of the mother’s first contact with the child.
3. In more than half of the cases, the first breastfeeding lasted as long as the specialists recommend – over 20 minutes.
